# Torsion of ovarian cyst during pregnancy: a case report

**DOI:** 10.1186/1757-1626-2-9405

**Published:** 2009-12-31

**Authors:** Vasavi Kolluru, Rekha Gurumurthy, Venkatasujatha Vellanki, Deshpande Gururaj

**Affiliations:** 1 Department of Obstetrics and Gynaecology, Kamineni Institute of Medical Sciences, Narketpally, Andhrapradesh, India-508254

## Abstract

In this case we report a 23 -year-old primigravida with 30 weeks presenting with torsion of the ovarian cyst. She presented to the antenatal clinic with acute pain abdomen. She was diagnosed to have torsion of ovarian cyst during pregnancy and a cystecomy was carried out. Her histopathology report showed a benign serous cystadenoma. Her pregnancy was followed up. She delivered a healthy female baby at term. Although the safety of antepartum surgical intervention has been accepted, abdominal surgery nevertheless carries some risks to a pregnant woman and unborn fetus, and so the choice of management necessitates a weighing of risks based on characterization of the adnexal mass and gestational age.

## Introduction

Torsion of ovary is the total or partial rotation of the adnexa around its vascular axis or pedicle. Moderate size, free mobility and long pedicle are predisposing factors. The exact etiology is obscure. Most commonly seen are dermoid and serous cystadenomas. Complete torsion causes venous and lymphatic blockade leading to stasis and venous congestion, haemorrhage and necrosis. The cyst becomes tense and may rupture. Patient usually presents with acute severe pain abdomen and pelvic examination may reveal a tender cystic mass separate from the uterus. The risk of ovarian torsion rises by 5 fold during pregnancy. Incidence is 5 per 10,000 pregnancies[1]. Torsion of ovarian tumors occurred predominantly in the reproductive age group. The majority of the cases presented in pregnant (22.7%) than in non-pregnant (6.1%) women[2].

## Case Report

23 year old primigravida presented to the antenatal clinic with 7 months amenorrhoea and pain abdomen and 3 episodes of vomitings, since one day. Her menstrual cycles were regular. She described the pain as sharp non-radiating type of pain in the right iliac fossa with sudden onset, with no relieving factors. She gave no history of vaginal bleeding or discharge. There was no history of diarrhoea, constipation, fever, urinary complaints or any recent illness. She conceived spontaneously. She had regular antenatal checkups. Her first & second trimesters were uneventful. No significant past medical and surgical history noted.

On examination, patient was conscious, coherent with pulse 82/min, blood pressure 130/80 mm of hg, temperature normal, cardiovascular and respiratory systems normal. Abdominal examination revealed fundal height corresponding to 30 weeks gestation. Uterus was irritable. There was a single fetus in longitudinal lie with breech presentation. Fetal heart rate was good & regular. There was severe tenderness in right iliac fossa. On vaginal examination, cervix was posterior, 50% effaced, 2 cm dilated, and breech presentation.

All her blood and urine investigations were within normal limits. Ultrasonography revealed a 10 × 5 cm single anechoic cystic lesion in right iliac fossa with single thin septation and no solid components. It also showed a single intrauterine live fetus in longitudinal lie with breech presentation of 30 wks gestational age. Estimated fetal weight-1629 gms, Amniotic fluid adequate, placental position posterior upper segment with grade 2 maturity. No evidence of free fluid in the abdomen.

**Figure 1:**
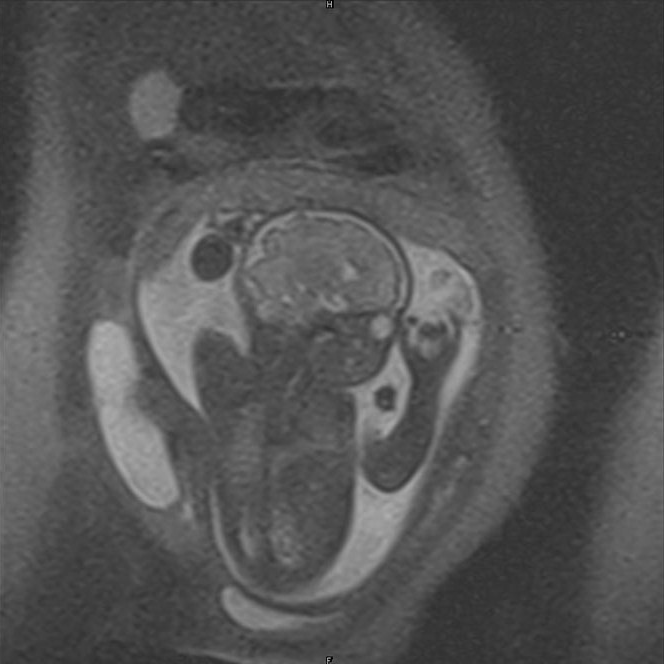
**Showing MRI image of ovarian cyst with fetus in utero**.

**Figure 2:**
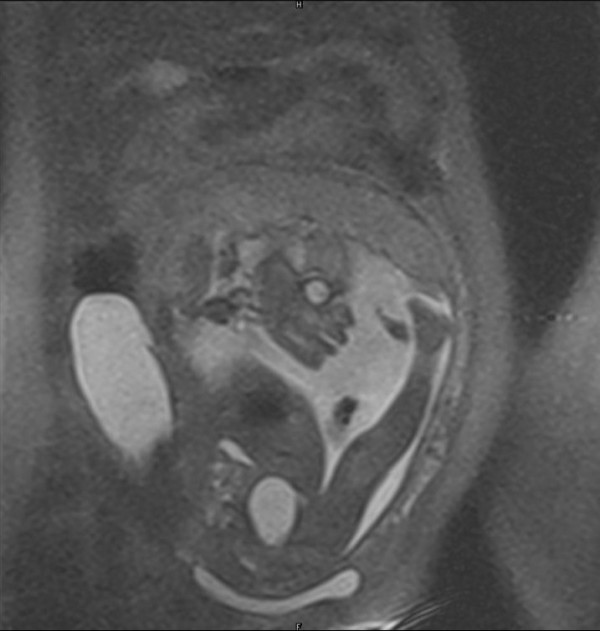
**MRI image of fetus with ovarian cyst**.

MRI showed extended breech presentation of the fetus and a large hyper intense mass lesion on the right side of the abdomen, outside the uterus measuring 10.4 × 5.0 cms suggestive of right ovarian cyst. (Figure 1, 2)

**Figure 3:**
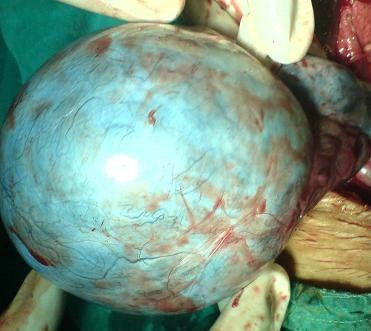
**Ovarian cyst showing torsion around its pedicle**.

With the provisional diagnosis of twisted ovarian cyst, emergency laparotomy was done under regional anaesthesia. A 10 × 5 cm right ovarian cyst was found to be twisted around its pedicle by 3 rotations. After untwisting, cystectomy was done by carefully enucleating the cyst and separating it from the capsule. The cyst was sent for histopathological examination. (Figure 3)

Patient recovered with an uneventful postoperative period and was discharged on 9^th ^post operative day. Her histopathology report showed benign serous cystadenoma of the ovary. She was followed up, her pregnancy continued unremarkably and she delivered an alive female baby of birth weight 2.75 kg at term gestation by caesarean section.

## Discussion

The commonest type of ovarian tumours encountered in pregnancy are cystic teratoma, paraovarian cyst, serous cystadenoma, corpus luteal cysts, luteomas etc[2]. Serous cystadenomas are thin walled, translucent cysts usually unilocular, may have few daughter cysts, varying between 20-30 cms in size. They are often unilateral can be bilateral.10-15% of them are borderline malignant while 20- 40% are malignant.

Differential diagnosis includes: uterine leiomyomas, non preganant horn of bicornuate uterus, appendiceal abscess, diverticular abscess, pelvic kidney, retroperitoneal tumours, ectopic pregnancy and retroverted gravid uterus[2].

Complications of the cysts associated with pregnancy are torsion of the cyst, rupture, infection, malignancy, impaction of cyst in pelvis causing retention of urine, obstructed labour and malpresentations of the fetus[2]. Some studies have suggested surgical intervention for concerns of malignancy, tumor torsion. Tumor rupture, or obstruction of labor[3,4]. Other studies have recommended the principle of observation, finding that most ovarian masses can either remain uneventful or resolve throughout pregnancy and that the incidence of the above risks was actually low[3,5]. Its most common cause in pregnancy is a corpus luteum cyst, which usually regresses spontaneously by the second trimester[6]. Ovarian torsion, therefore, occurs most frequently in the first trimester, occasionally in the second, and rarely in the third[7].

## Management

Cysts less than 6 centimetres in diameter and appearing benign on ultrasound are generally treated conservatively as they may undergo spontaneous resolution. Corpus luteal cysts regress by 12 to 16 weeks. Cysts more than 10 centimetres in size are usually resected due to increased risk of malignancy, rupture or torsion. Management of cysts between 5 to 10 centimetres is controversial. If the cysts contain septae, nodules, papillary excrescences or solid components then resection is recommended. Those with simple cystic appearance may be managed expectantly with serial ultrasound surveillance. However they may require emergency exploratory laparotomy for rupture, torsion or infarction in as many as 50% cases [3]. With the advent of imaging techniques like high resolution ultrasound, MRI and transvaginal colour Doppler, the expectant management has become much more common.

If the ovarian cyst is diagnosed in the first trimester, it is better to wait till 16 wks when the implantation of pregnancy is more secure and also the cyst may disappear spontaneously. Persisting tumours are treated by cystectomy or ovariotomy as indicated. Ovarian tumour or cyst can be easily removed till 28 wks of gestation thereafter it is not readily accessible and may precipitate preterm labour. Ovarian cyst which ruptures, or undergoes torsion or if it shows evidence of malignancy, requires immediate surgery, irrespective of the period of gestation [3].

A simple cystectomy can be performed in the absence of overt malignancy. Previously untwisting of the pedicle was avoided to prevent emboli and toxic substances related to hypoxia, from entering peripheral circulation. but recently, re-establishing ovarian circulation by untwisting, has shown to result in viable ovarian tissue with no systemic complications[1].

## Conclusion

Ovarian torsion is relatively uncommon in the second trimester of pregnancy. Diagnosis can usually be made on the basis of the characteristic clinical presentation in conjunction with ultrasound evidence of a unilaterally enlarged adnexal mass. Treatment options are limited to surgery, either by laparoscopy or laparotomy, but the former becomes more difficult after second trimester.

## Consent

Written informed consent was obtained from the patient for publication of this case report. A copy of the written consent is available for review by the Editor-in-Chief of this journal.

## Competing interests

The authors declare that they have no competing interests.

## Authors' contributions

The case was managed and operated by RG, VK, and VVS. The authors have read and approved the final manuscript.
